# SN50 attenuates alveolar hypercoagulation and fibrinolysis inhibition in acute respiratory distress syndrome mice through inhibiting NF-κB p65 translocation

**DOI:** 10.1186/s12931-020-01372-6

**Published:** 2020-05-27

**Authors:** Yanqi Wu, Yahui Wang, Bo Liu, Yumei Cheng, Hong Qian, Huilin Yang, Xiang Li, Guixia Yang, Xinghao Zheng, Feng Shen

**Affiliations:** 1 Department of Critical Care Medicine, Guizhou Medical University Affiliated Hospital, Guiyang, 550001 China; 2 Guizhou Medical University, Guiyang, 550001 China; 3 The People’s Hospital of Weining County, Bijie, 553100 Guizhou Province China

**Keywords:** SN50, Acute respiratory distress syndrome, Alveolar hypercoagulation, fibrinolysis inhibition.

## Abstract

**Background:**

It has been confirmed that NF-κB p65 signaling pathway is involved in the regulation of alveolar hypercoagulation and fibrinolysis inhibition in acute respiratory distress syndrome (ARDS). Whether SN50, a NF-κB cell permeable inhibitor, could attenuate alveolar hypercoagulation and fibrinolysis inhibition in ARDS remains to be elucidated.

**Purpose:**

We explored the efficacy and potential mechanism of SN50 on alveolar hypercoagulation and fibrinolysis inhibition in ARDS in mice.

**Materials and methods:**

Mouse ARDS was made by 50 μl of lipopolysaccharide (LPS) (4 mg/ml) inhalation. Male BALB/c mice were intraperitoneally injected with different does of SN50 1 h before LPS inhalation. Lung tissues were collected for hematoxylin-eosin (HE) staining, wet/dry ratio. Pulmonary expressions of tissue factor (TF), plasminogen activator inhibitor-1 (PAI-1), collagen III, as well as phosphorylated p65 (p-p65), p65 in nucleus (p’-p65), IκBα and IKKα/β were measured. Bronchoalveolar lavage fluid (BALF) was gathered to test the concentrations of TF, PAI-1, activated protein C (APC) and thrombinantithrombin complex (TAT). DNA binding activity of NF-κB p65 was also determined.

**Results:**

After LPS stimulation, pulmonary edema and exudation and alveolar collapse occured. LPS also stimulated higher expressions of TF and PAI-1 in lung tissues, and higher secretions of TF, PAI-1, TAT and low level of APC in BALF. Pulmonary collagen III expression was obviously enhanced after LPS inhalation. At same time, NF-κB signaling pathway was activated with LPS injury, shown by higher expressions of p-p65, p’-p65, p-IKKα/β, p-Iκα in pulmonary tissue and higher level p65 DNA binding activity. SN50 dose-dependently inhibited TF, PAI-1 and collagen IIIexpressions, and decreased TF, PAI-1, TAT but increased APC in BALF. SN50 treatment attenuated pulmonary edema, exudation and reduced lung tissue damage as well. SN50 application significantly reduced p’-p65 expression and weakened p65 DNA binding activity, but expressions of p-p65, p-IKKα/β, p-Iκα in cytoplasm of pulmonary tissue were not affected.

**Conclusions:**

SN 50 attenuates alveolar hypercoagulation and fibrinolysis inhibition in ARDS via inhibition of NF-κB p65 translocation. Our data demonstrates that NF-κB p65 pathway is a viable new therapeutic target for ARDS treatment.

## Background

Acute respiratory distress syndrome (ARDS), induced by many pathogenic factors, such as pneumonia, sepsis, shock etc., is one of the most common causes being treated in ICU. It is characterized by respiratory distress and progressively refractory hypoxemia [1–4]. Although protective ventilation, conservative fluid management, extracorporeal membrane oxygenation (ECMO) and some other supporting therapies improved its clinical outcome, the mortality of ARDS remains as high as 30–50% [5]. Hypercoagulation and fibrinolysis inhibition in airspace is a critical pathophysiology [6], which are the important reasons responsible for the high mortality of ARDS. Alveolar hypercoagulation and fibrinolysis inhibition contribute to microthrombus formation in pulmonary vessels and fibrin deposits in airspace, which are associated with imbalance of V/Q ratio, decreased lung compliance, diffusion disorder, etc., resulting in refractory hypoxemia and pulmonary fibrosis [7, 8]. Our previous studies confirmed that NF-κB signaling pathway participated in the regulation of hypercoagulation and fibrinolysis inhibition in LPS-induced alveolar epithelial cell type II (ACEII) [9.10].

Nuclear factor kappa B (NF-κB) is a ubiquitous transcriptional factor participating in regulation of immune and inflammatory responses [11]. The mammalian NF-κB family consists of p65, c-Rel, RelB, p50 and p52, which exist in the resting state as homodimers or heterodimers primarily bound to their inhibitory protein IκBs under physiological conditions, and p65 is the main transcriptional factor. Once NF-κB signaling pathway being activatied, IκBs is degraded by the IκB kinase complex (IKKBs), unmasking the nuclear localization sequence of NF-κB and allowing NF-κB dimer to translocate into nucleus, where NF-κB binds to the promoter and enhancer regions of its target genes containing κB sites, resulting in genes transcription [12–14]. In previous experiments, we found that silencing NF-κB p65 gene or regulating IKKβ modulated the LPS-stimulated expressions of TF, PAI-1 and APC in alveolar epithelial cell type II (AECII) [9, 10].

SN50, the NF-κB cell permeable inhibitory peptide, was first synthesized by Lin et al. in 1995 [15]. It was comprised of the hydrophilic region of the signal peptide of Kaposi fibroblast growth factor as a membrane translocating motif and a nuclear localization sequence derived from the p50 subunit of NF-κB [15]. Chian et al. showed that SN50 protected against LPS-induced lung injury in isolated rat lung by inhibiting NF-κB nuclear translocation [16]. Based on that finding, we speculate that SN50 would correct alveolar coagulation and fibrinolysis abnormalities via NF-κB signaling pathway in ARDS. So we tested the effects and the mechanism of SN50 on alveolar hypercoagulation and fibrinolysis inhibition in LPS-induced mouse ARDS.

## Materials and methods

### Animal preparation

The study was performed in accordance with animal ethics guidelines of Guizhou Medical University. Briefly, male Balb/c mice, aged 8 to 12 weeks and weighing 20 ± 2 g, were obtained from the laboratory animal center at Guizhou Medical University. The whole experiment performed in this study was conformed to the Guide for the Care and Use of Laboratory Animals and were approved by the Institutional Animal Care and Use Committee.

### Experimental protocols

The mice were randomly divided into 6 experimental groups: control, N-SN50, LPS, L-SN50, M-SN50 and H-SN50 group, with 12 mice in each. Mice in control and LPS group received 100 μl of PBS intraperitoneal injection. Mice in N-SN50 and M-SN50 group received 100 μl of SN50 with concentration of 30 μg/ml, while mice in L-SN50 and H-SN50 group received same amount of SN50 with concentration of 10 μg/ml and 60 μg/ml respectively. An hour later of intraperitoneal injection of SN50, mice were anaesthetized with intraperitoneal injection of chloral hydrate and then were fixed on the operating table with supine position, followed by 50 μl of LPS (4 mg/ml) being sprayed into the trachea of mice in LPS, L-SN50, M-SN50, and in H-SN50 groups by using an aerosol endotracheal drug suit (Yuyan Instruments, Shanghai, No.YAN30012), but LPS was substituted by PBS in control and N-SN50 groups. Six hours later the mice were euthanized and lung tissue and BALF were collected under anesthetization.

### BALF collection

After 6 h of LPS aspiration, a tracheotomy was performed and a catheter was inserted into the trachea. One milliliter of sterile PBS buffer was slowly injected into the trachea with a sterile syringe, and the PBS was suctioned out as much as possible 30 s later. PBS injection was repeated for three times. BALF were harvested and stored at − 80 °C for testing.

### Haematoxylin and eosin staining

The right lower lobe of lung was embedded in paraffin and sagittally sliced at 5 μm. The sections were stained with haematoxylin and eosin. Oedema, alveolar and interstitial inflammation and haemorrhage, atelectasis, necrosis, and hyaline membrane formation were applied for lung injury scoring under microscope (0, no injury; 1, injury in 25% of the field; 2, injury in 50%; 3, injury in 75%; and 4, injury throughout the field). The total lung injury score was calculated as the sum of these scores. Ten randomly selected high-power fields (400×) in each slide were analyzed by two investigators who were blinded to the mouse groups.

### Evaluation of the lung oedema

Briefly, the whole lung was removed and cleared of all extra pulmonary tissues and the wet weight was recorded. Then the lung tissue was placed in a clean container and baked in a constant temperature oven at 65 °C for 72 h until the weight didn’t change any more, and recorded the dry weight.

### Western immunoblot analysis

Total protein lysates were extracted from lung tissues using RIPA lyses buffer. The lung tissues were homogenized in PBS containing the protease inhibitor cocktail. The homogenates were centrifuged at 14, 000 rpm in 4 °C for 15 min. Supernatants of lung tissues were collected and protein concentration was measured using BCA protein assay kit (Solarbio Life Sciences, Beijing). An equal amount of protein from each sample was resolved in 10% Tris-glycine SDS polyacrylamide gel. Protein band was blotted to nitrocellulose membrane. After incubation for 1 h in blocking solution at room temperature, the membrane was incubated for 24 h with anti-TF (1:500 Santa Cruz Biotechnology, Inc.), anti-PAI-1 (1:500 Santa Cruz Biotechnology, Inc.), anti-β-actin (1:1000 Santa Cruz Biotechnology, Inc.), anti-p-IKKα/β (1:800 Santa Cruz Biotechnology, Inc.), anti- p-IκBα (1:800 Santa Cruz Biotechnology, Inc.), anti-p65 (1:800 Santa Cruz Biotechnology, Inc.), anti- p-p65(1:800 Santa Cruz Biotechnology, Inc.), at 4 °C. The secondary antibody (horseradish peroxidase-conjugated donkey anti-rabbit immunoglobulin) was added at 1:10,000 dilution and incubated at room temperature for 1 h. Peroxidase labeling was detected with the enhanced chemiluminescence Western blotting detection system (Amersham Pharmacia Biotech) and analyzed by a densitometry system. The relative protein level was normalized to β-actin (Abcam Biotechnology, Inc.).

### Quantitative real time RT-PCR analysis

Total RNA was isolated using an RNeasy Mini kit including DNase I digestion enzyme. Following reverse transcription, quantitative real-time PCR analysis was performed using the ABI ViiATM 7 real-time PCR system (Thermo Fisher Scientific) with SYBR Green master mix. The primers used for analysis were synthesized by Shanghai Sangon Biotech (Table 1). We performed PCR amplification with cDNA being used as template. The reaction system was set as follows: SYBR Green Mix 10 μl, forward primer and reverse primer 0.8 μl respectively, cDNA template 0.8 μl, ddH2O 7.6 μl, which were synthesized into a system containing 20 μl reagents. Dissolution and amplification curve of the gene were recorded following the gene amplification. Expressions of target genes were calculated using the 2-ΔΔCt method.

**Table 1:** Primer sequences of each gene

Gene	Forward(5’-3’)	Reverse(3’-5’)	Primer Length (bp)
TF	AATGGGCAGATAGAGTGT	ACCGCATTCAAGTCA	182
PAI-1	ACCAACTTCGGAGTAAAA	TTGAATCCCATAGCATCT	158

### ELISA assay

TF, PAI-1, APC and TAT levels in BALF were determined by using ELISA kits (Jiyinmei Bio-company, Wuhan, China) according to the manufacturer’s instructions.

### NF-κB p65 DNA binding activity assay

For detecting NF-κB p65 DNA binding activity, TransAMTM NF-κB p65 Chemi Transcription Factor Assay Kit was used (Active Motif, Carlsbad, CA).

### Immunohistochemistry

Lung slides were deparaffinized and rehydrated in xylene and ethanol. After antigen retrieval, the lung slides were treated with 0.3% H_2_O_2_ in methanol for 30 min and subsequently incubated with blocking solution (5% goat serum) for 20 min at RT. The lung slides were incubated with anti-collagen III (1:200) for 90 min at RT. After washing three times in PBS, the lung slides were incubated with secondary antibody (1:500) labelled with polymer and horseradish peroxidase (Solarbio Life Sciences, Beijing) for 30 min at RT. The immunoreactions were visualized using a diaminobenzidine substrate kit (Dako, Carpinteria, CA). The lung slides were mounted with malinol (Muto Pure Chemicals, Tokyo, Japan). Signals were classified into 4 grades of intensity as follows: (−) negative; (+) weakly positive, (++) moderately positive, and (+++) strongly positive.

### Statistical analysis

Values are represented as mean ± SEM of at six independent experiments. Statistical analysis was performed using SPSS 24.0 (IBM Corporation, Armonk, NY, USA). ANOVA and independent Student’s t-tests were used to analyze the differences between the different groups. *p* < 0.05 was considered statistically significant.

## Results

### SN50 attenuated pulmonary inflammation and pulmonary edema in LPS-induced ARDS mice

**Fig. 1:**
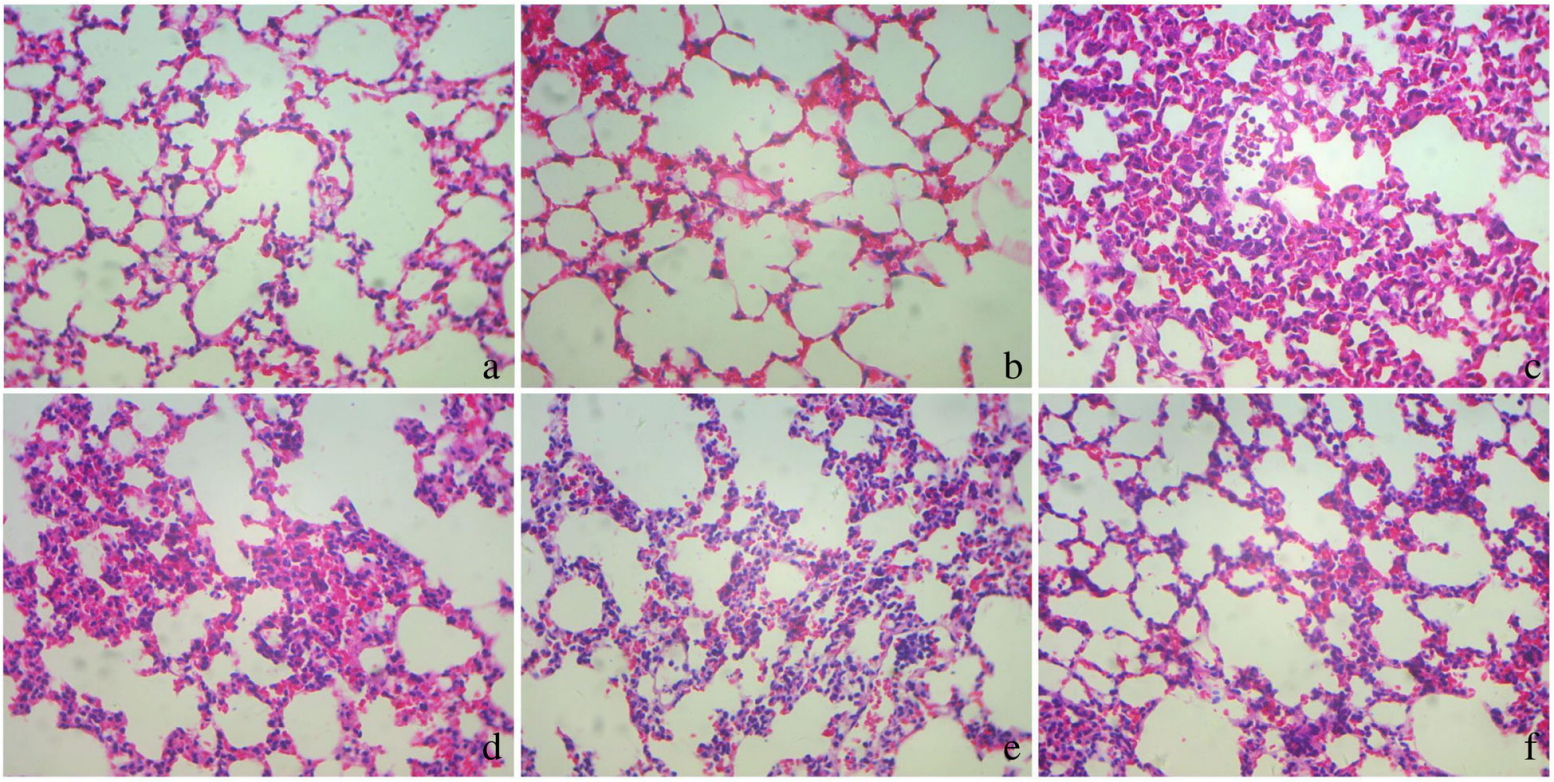
Pathological changes in the lung tissues. 6 h after LPS inhalation, mice were exsanguinated and their right lower lungs were fixed. Then, tissue sections were stained with hematoxylin and eosin. The figure demonstrates a representative view (× 100) from each group, showing that LPS destroyed the lung tissue while SN50 prenvented lung tissue from injury, in which with concentration of SN50 increasing, the protective role became more obvious (**a**: Control, **b**: N-SN50, **c**: LPS, **d**: L-SN50, **e**: M-SN50, **f**: H-SN50)

**Fig. 2:**
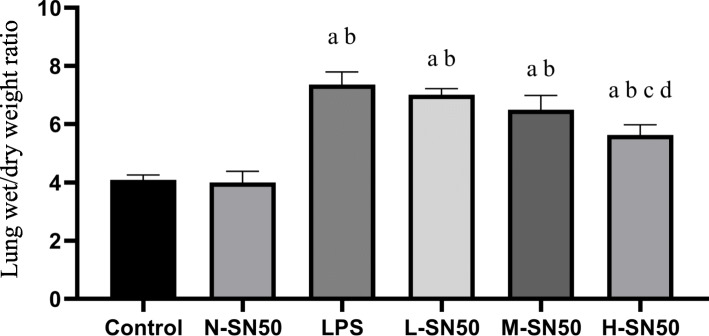
Lung wet/dry weight ratio. Each bar represents the mean ± SD. Compared to control group (4.097 ± 0.163), W/D ratio in LPS group increased (7.360 ± 0.434). Application of SN50, however, dose-dependently decreased W/D ratio, with (7.010 ± 0.211) in L-SN50 group, (6.493 ± 0.497) in M-SN50 group and (5.630 ± 0.352) in H-SN50 group respectively. ^a^p<0.05 compared with Control. ^b^p<0.05 compared with N-SN50. ^c^p<0.05 compared with LPS. ^d^p<0.05 compared with L-SN50

ARDS mice showed obvious exudation,inflammatory cells infiltration, alveolar fibrin deposition and alveolar collapse. SN50 attenuated these changes in dose-dependent manner (Fig. 1). ARDS mice also experienced severe lung injury and pulmonary edema, which prevented by pretreatment with SN50, also in dose-dependent manner (Fig. 2).

### SN50 inhibited expressions of TF and PAI-1 in mRNA and in protein levels in ARDS mice

**Fig. 3:**
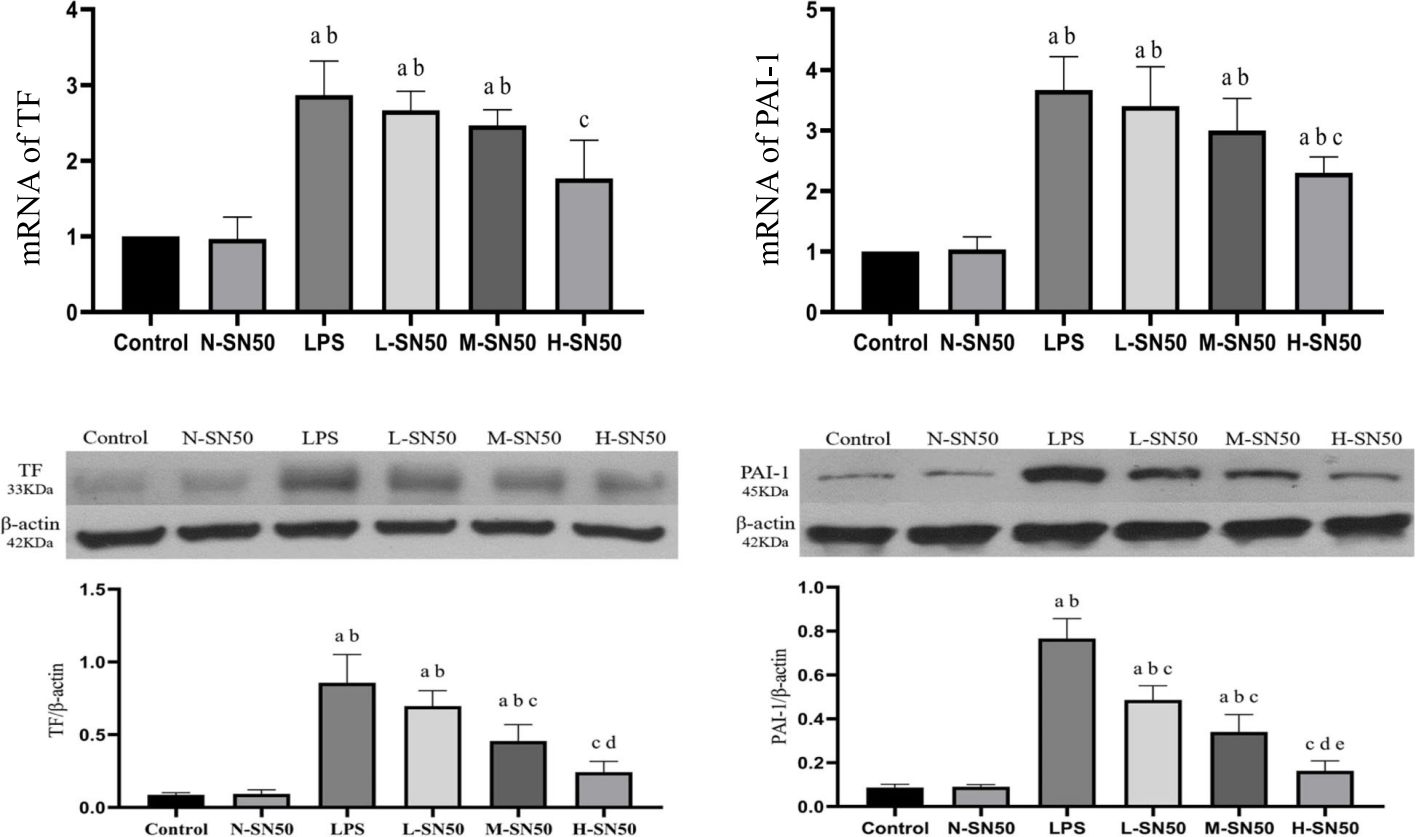
mRNA and protein expressions of TF and PAI-1 in lung tissue. Each bar represents the mean ± SD. Compared to control and N-SN50 group, expressions of TF and PAI-1 were higher in LPS group. L-SN50, M-SN50 and H-SN50 showed decreasing trends of the expressions. mRAN expression appeared the same trend changes as protein levels did in each gruop. ^a^p<0.05 compared with Control. ^b^p<0.05 compared with N-SN50. ^c^p<0.05 compared with LPS. ^d^p<0.05 compared with L-SN50. ^e^p<0.05 compared with M-SN50

To determine whether SN50 modulates TF and PAI-1 expressions in lung tissue of LPS-induced ARDS, we detected these mRNA and protein expressions by quantitative real-time PCR (qRT-PCR) and western blotting respectively. LPS inhalation up-regulated TF and PAI-1 expressions either in mRNA or in protein in ARDS mice. However, SN50 injection downregulated these expressions in dose-dependent manner. (Fig. 3).

### SN50 inhibited secretions of TF, PAI-1, TAT and promoted APC production in BALF in mice with ARDS

**Fig. 4:**
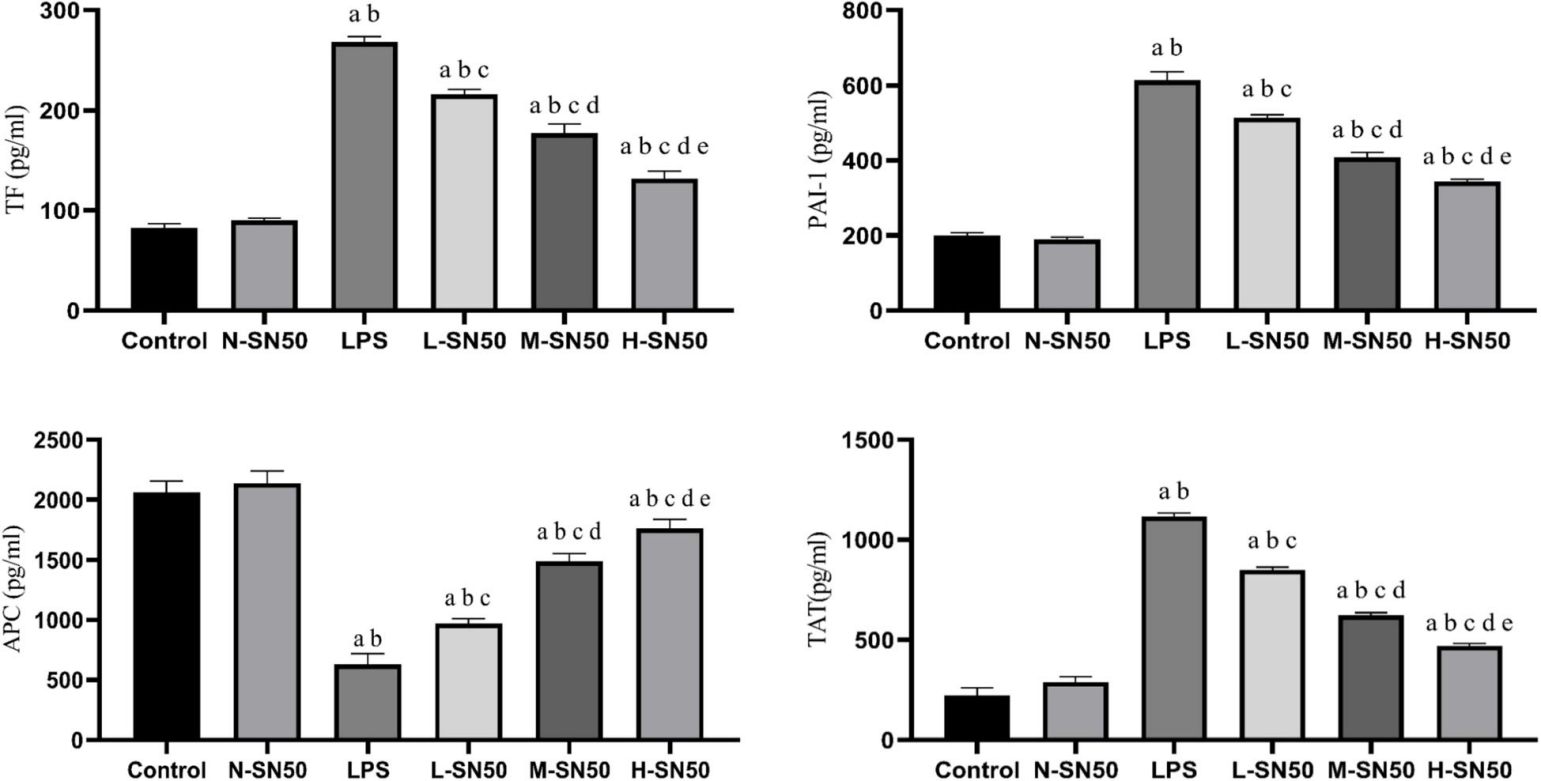
SN50 reversed TF, PAI-1, APC and TAT secretions in BALF induced by LPS. Each bar represents the mean ± SD. TF, PAI-1 and TAT were substantially released from lung tissue into BALF while APC secretion was depressed under LPS stimulation. But SN50 significantly reversed the effects of LPS in each group. ^a^p<0.05 compared with Control. ^b^p<0.05 compared with N-SN50. ^c^p<0.05 compared with LPS. ^d^p<0.05 compared with L-SN50. ^e^p<0.05 compared with M-SN50

We also collected BALF to measure the concentrations of TF, PAI-1, TAT and APC by enzyme-linked immunosorbent assay (ELISA), so as to determine the effects of SN50 on alveolar coagulation and fibrinolytic inhibition. The results indicated that LPS stimulated substantial release of TF, PAI-1 and TAT and less APC production, which were all dose-dependently reversed by SN50 pretreatment. (Fig. 4).

### SN50 attenuated the high expression of collagen III induced by LPS in lung tissues

**Fig. 5:**
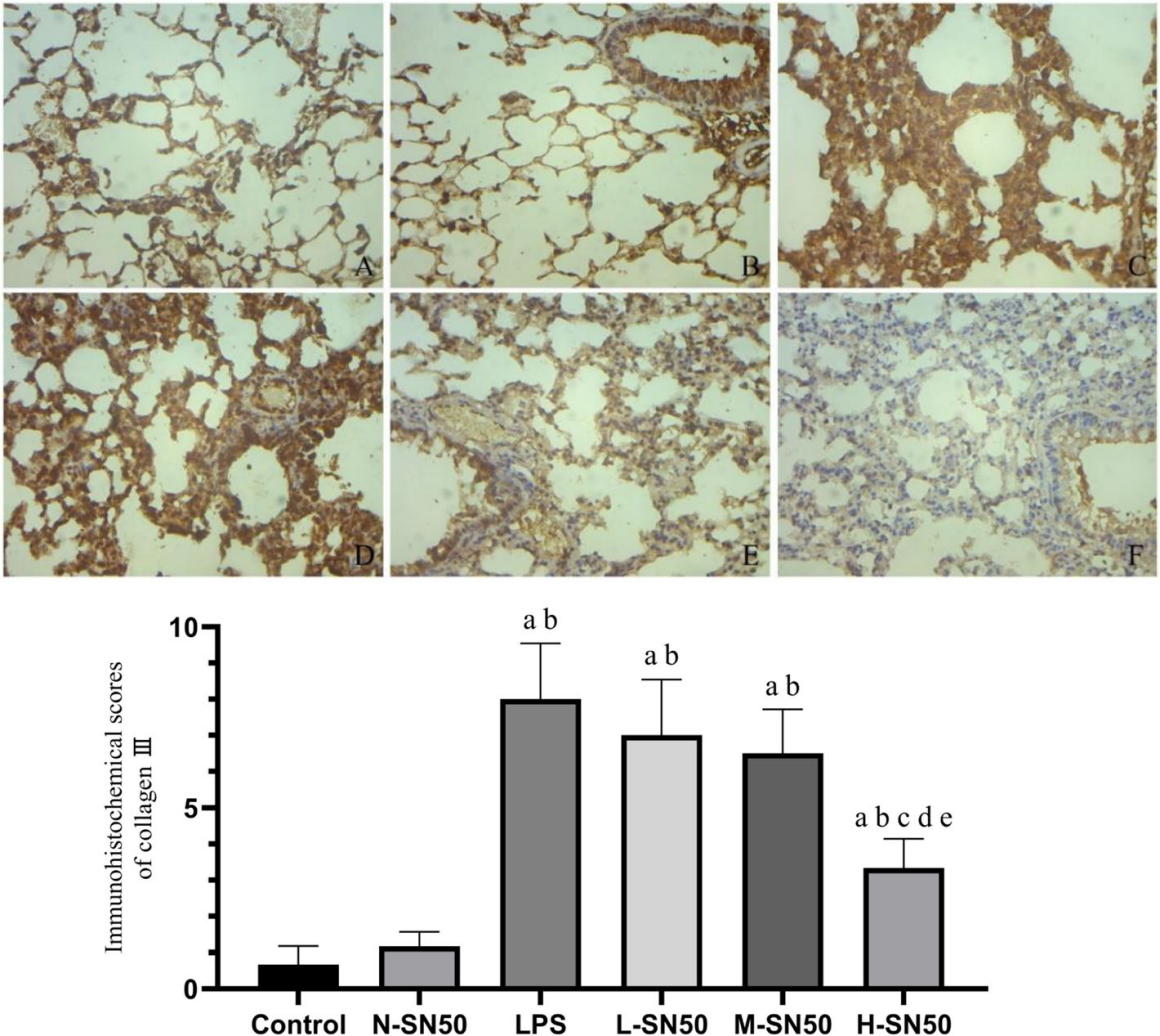
Collagen III expression in lung tissue and its immunohistochemical scores The figure demonstrates a representative view (× 100) from each group. Each bar represents the mean ± SD. The LPS group showed deepest staining, indicating that collagen III expression was significantly increased. Following with SN50 intervention, collagen III expression decreased obviously and the staining faded. ^a^p<0.05 compared with Control. ^b^p<0.05 compared with N-SN50. ^c^p<0.05 compared with LPS. ^d^p<0.05 compared with L-SN50. ^e^p<0.05 compared with M-SN50. (A, Control group; B, N-SN50; C, LPS group; D, L-SN50, E, M-SN50, F, H-SN50).

In order to check the degree of pulmonary fibrosis provoked by LPS, we measured the collagen III expression by immunohistochemistry. LPS stimulation promoted the collagen III expression in pulmonary tissue (shown by dark tan, Fig. 5). SN50 pretreatment, however, decreased collagen III expression, the higher the doses of SN50, the more obvious the decrease.

### SN50 did not affect expressions of p-IKKα/β, p-IκBα and p-p65 in lung tissue

**Fig. 6:**
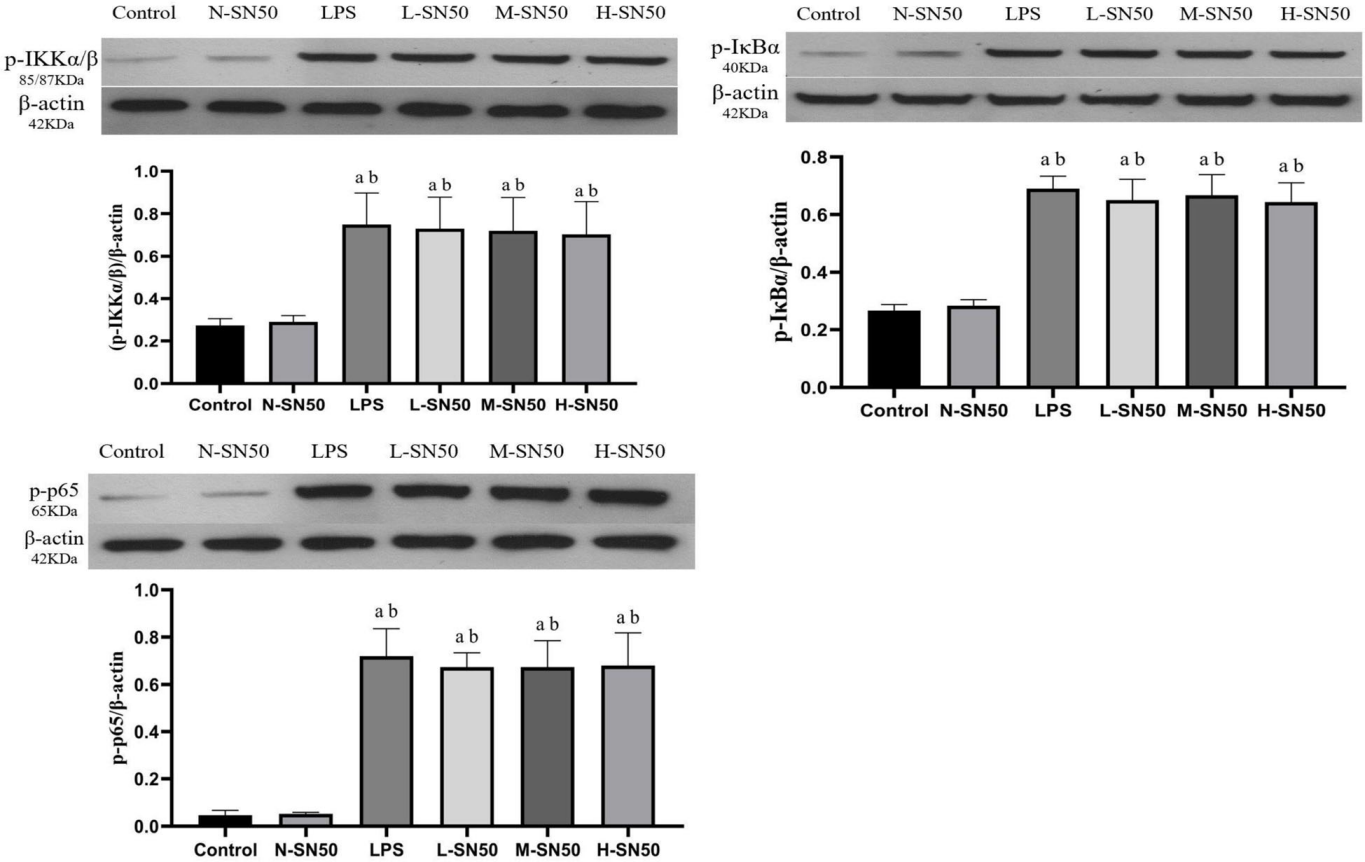
Protein expressions of p-IKKα/β, p-Iκα and p-p65 in lung tissues. Each bar represents the mean ± SD. Compared to control group, expressions of p-IKKα/β, p-IκBα and p-p65 were all increased significantly in LPS group. However, these expressions in L-SN50, M-SN50 and H-SN50 group did not show any differences with those in LPS group. ^a^p<0.05 compared with Control. ^b^p<0.05 compared with N-SN50

To understand whether the efficacy of SN50 on pulmonary coagulation and fibrinolysis in ARDS is related with activation of the NF-κB pathway, we determined the p-IKKα/β, p-IκBα and p-p65 expressions in lung tissue by western blotting. Results showed that LPS stimulation resulted in a higher expressions of p-IKKα/β, p-IκBα and p-p65 in lung tissue, indicating NF-κB pathway activation, but SN50 application did not affect these expressions at all. (Fig. 6).

### SN50 blocked the translocation of phospho-p65 from cytoplasm to nucleus and decreased p65 DNA binding activity initiated by LPS stimulation

**Fig. 7:**
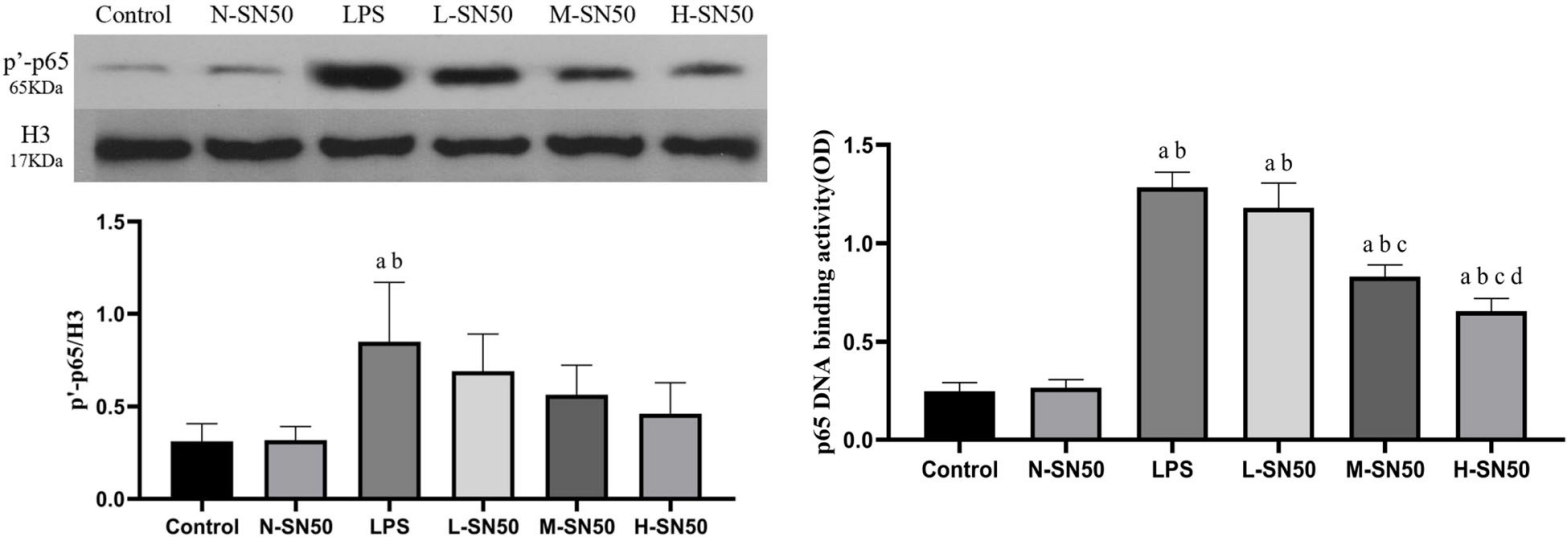
Nuclear translocation of p-p65 and p65 DNA binding activity. Each bar represents the mean ± SD. p-p65 level in nucleus was measured by western-blotting (A), and p65 DNA binding activity was determined by EILSA (B). Compared to control group, both expressions of p-p65 in nuclear and p65 DNA binding activity were significantly increased in LPS group. SN50 treatment decreased the p-p65 expression and weakened p65 DNA binding activity, indicating the inhibiting of translocation of p-p65 from cytoplasm to nuclear. ^a^p<0.05 compared with Control. ^b^p<0.05 compared with N-SN50. ^c^p<0.05 compared with LPS. ^d^p<0.05 compared with L-SN50

To understand whether SN50 blocks the translocation of p-p65 from cytoplasm to nucleus and whether SN50 affects the transcriptional activity or not, p-p65 protein expression in nucleus and p65 DNA binding activity were determined by western blotting and by ELISA respectively. LPS stimulation promoted nuclear p-p65 expression and increased p65 DNA binding activity as well, while SN50 dose-dependently decreased the p-p65 expression in nucleus, indicating blockage of p-p65 translocation from cytoplasm to nucleus, and also weakened p65 DNA binding activity either. (Fig. 7).

## Discussion

Our results showed that LPS-induced lung injury experienced pulmonary edema, lung tissue deconstruction, high level pulmonary expressions of TF and PAI-1, large amount of secretions of TF, PAI-1, TAT and decreased APC production in air space, and a high content of pulmonary fiber (high collagen III), all which indicated alveolar hypercoagulaiton and fibrinolysis inhibition. Meanwhile, NF-κB pathway was activated with an increased p65 translocation from cytoplasm to nucleus and enhanced p65 DNA binding activity. Our results were consistent with published data by Chian et al. [16]. SN50 treatment not only down-regualted TF and PAI-1 expressions in pulmonary tissue, but also inhibited secretions of TF, PAI-1 and TAT and decreased pulmonary collagen III level, and promoted APC production in BALF, demonstrating that SN50 effectively corrected hypercoagulation and fibrinolysis inhibition in LPS-induced ARDS. In exploring the mechanism of SN50, we found that SN50 decreased p65 level in nucleus and decreased p65 DNA binding activity but with no influence on p-IKKα/β, p-IκBα and p-p65 in lung tissue. Therefore, we have the reason to think that SN50 corrected hypercoagulation and fibrinolysis inhibition by preventing p65 translocation from cytoplasm into nuleus and inhibiting p65 DNA binding capacity rather than other mechanisms. Our data showed that the changes of TF, PAI-1, and of some other procoagulant factors and fibrinolytic inhibitors in BALF, as well as of lung injuries and pulmonary edema did not reach the baseline in SN50- treated mice, which implies that SN 50 partially attenuated but not abrogated LPS-induced ARDS.

SN50 is a cell-permeable peptide which can specifically inhibit the NF-κB p65 translocation [17, 18]. Previous studies have demonstrated that it can inhibit inflammatory cells infiltration and protect rat lung against LPS induced-lung injury, attenuating traumatic brain injury in mice [16, 19]. As far as we know, this is the first to explore the effect of SN50 on hypercoagulation and fibrinolysis inhibition induced by LPS.

NF-κB activation could be either by canonical or noncanonical pathways. Studies demonstrated that there were several methods to inhibit NF-κB pathway, including inhibition of IκB kinase complex [9, 10, 20], decrease of IκB protein degradation [21], etc. In our study, we noticed that SN50 treatment resulted in reduction of translocation of p65 from cytoplasm to nucleus and attenuation of p65 DNA binding activity, while p-IKKα/β, p-p65, p-Iκα kept unchanged, indicating that SN50 specifically inhibited p65 translocation from cytoplasm to nucleus and decreased p65 DNA binding activity. Experiments in vivo and in vitro have confirmed that NF-κB signal pathway was involved in many pathologic process such as anti-inflammatory response in sepsis [22–24], downregulation of cytokines and MAPK activation in LPS-induced lung injury [16]. Our previous studies confirmed NF-κB p65 also participated in regulation of LPS-induced hyperexpressions of procoagulant factor TF and fibrinolysis inhibitor PAI-1 in alveolar epithelial cell type ‖, demonstrating it was associated with alveolar hypercoagulation and fibrinolysis inhibition in ARDS. In present animal study, our data demonstrated SN50 effectively down-regulated TF and PAI-1 expressions in lung tissue, decreased concentrations of procoagulants (TF, PAI-1, TAT) and promoted activated protein C (APC) in BALF, and attenuated collagen III expression in pulmonary tissue, suggesting that SN50 is expected to be an effective target to attenuate hypercoagulation and fibrinolysis inhibition in ARDS.

Our data indicated that NF-κB was involved in regulation of hypercoagulation and fibrinolysis inhibition in ARDS. So it is pivotal to effectively block the NF-κB pathway, such that correct the abnormalities of coagulation and fibrinolysis in airspace in ARDS. As mentioned above, NF-κB pathway could be activated either by canonical or by noncanonical pathway, but whether it is canonical or noncanonical pathway, the translocation process of p65 from cytoplasm to nucleus is the common way through which NF-κB exerts its transcriptional role [12, 25]. Our results showed that SN50 treatment induced a decrease NF-κB p65 nuclear content in lung tissue and an attenuation in p65 DNA binding activity, indicating the potential protective valuability of SN50. Our results also demonstrated that the efficacies of SN50 on alveolar hypercoagulation and fibrinolysis inhibition and the lung protection role were related with the dosage of SN50, in that in a certain range, with the increase of dose, the effect of SN50 becomes more obvious., But whether there is a more optimal dose point of SN50 or not is worth further study.

There are some limitations in our experiment. First, there was no arterial blood gas analysis making the diagnosis of ARDS insufficient. Second, we took different doses of SN50 for pretreatment in LPS-induced mice just according to some previous published article [16, 26–28], but the optimal dose of SN50 to attenuate hypercoagulation and fibrinolysis inhibition remains to be elucidated, and so did for the optimal timing of SN50 administration. However, NF-κB was an important therapeutic target for the alveolar hypercoagulation and fibrinolysis inhibition and SN50 has been proven to effectively attenuate these abnormalities.

## Conclusions

Our data demonstrate that LPS-induced ARDS possessed alveolar hypercoagulation and fibrinolysis inhibition. SN50 dose-dependently attenuates the abnormalities of alveolar coagulation and fibrinolysis via inhibition of NF-κB p65 translocation from cytoplasm to nucleus and p65 DNA binding activities in LPS-induced ARDS.

## Data Availability

We could offer the data and material if there is any requirement.

## Abbreviation

*ARDS*: *acute respiratory distress syndrome*
*HE*: *hematoxylin-eosin*
*TF*: *tissue factor*
*PAI-1*: *plasminogen activator inhibitor-1*
*BALF*: *bronchoalveolar lavage fluid*
*APC*: *activated protein C;*
*TAT*: *thrombinantithrombin complex*
*ECMO*: *extracorporeal membrane oxygenation*
*AECII*: *alveolar epithelial cell type II*
*LPS*: *lipopolysaccharide*
*ELISA*: *enzyme-linked immunosorbent assay*
